# Supplementary material for: A Systematic Review of Aperiodic Neural Activity in Clinical Investigations
Source: Eur J Neurosci. 2025 Oct 11;62(7):e70255. doi: 10.1111/ejn.70255 (PMC12514750; doi:10.1111/ejn.70255)
Supplement: Supplementary file 1 — Appendix S1: Literature Search Terms. [file EJN-62-0-s001.docx]

**Appendix: Literature Search Terms**

The following details the search terms used for the literature collection. For further details on how these terms were used, see the Methods section. For the use of these search terms in automated literature searches, see the Project Repository.

**Aperiodic activity search terms (used for all searches)**:

'aperiodic exponent', 'aperiodic slope', 'spectral exponent', 'spectral slope', '1/f slope', '1/f exponent'

**Phase 1 search terms for clinically related reports on aperiodic activity**:

'clinical', 'disorder', 'disease', 'biomarker', 'diagnosis', 'diagnostic', 'treatment'

**Phase 2 search terms for reports per disorder**:

"parkinson's"; 'epilepsy', 'seizure'; 'ADHD', 'attention deficit hyperactivity disorder'; 'autism', 'ASD'; 'alzheimers', 'dementia'; 'disorders of consciousness', 'coma', 'locked-in'; 'depression', 'MDD', 'major depressive disorder'; 'schizophrenia'; 'stroke'; 'dystonia'; 'TBI', 'traumatic brain injury'; 'dyslexia'; 'glioma'; "huntington's"; 'multiple sclerosis'; 'PTSD', 'post traumatic stress disorder'; 'REM sleep behavior disorder'; 'rett syndrome'; '22q.11.2'; 'ALS', 'amyotrophic lateral sclerosis', "Lou Gehrig's disease"; 'anxiety'; 'CDKL5 deficiency disorder'; 'chronic pain'; 'concussion'; 'down syndrome'; 'fibromyalgia'; 'fragile X'; 'insomnia'; 'NF1'; 'NREM parasomina'; 'OCD', 'obsessive compulsive disorder'; 'STXBP1'; 'tinnitus'; 'tourette'; 'tuberous sclerosis complex'; ‘delirium’; ‘stutter’; ‘cancer’

**Exclusion terms, used to ignore unrelated literature**:

'acid', 'protein', 'ion', 'enzyme', 'ultrasound', 'cancer', 'halide', 'spectroscopy', 'iodide', 'tissue'
